# Aberrant courses of the occipital artery

**DOI:** 10.1007/s00276-025-03650-8

**Published:** 2025-04-30

**Authors:** Mugurel Constantin Rusu, Cătălin Constantin Dumitru, Răzvan Costin Tudose

**Affiliations:** 1 Division of Anatomy, Department 1, Faculty of Dentistry, “Carol Davila” University of Medicine and Pharmacy, 8 Eroilor Sanitari Blvd, Bucharest, RO 050474 Romania; 2 Research Department, “Dr. Carol Davila” Central Military Emergency Hospital, Bucharest, -010825 Romania

**Keywords:** Digastric muscle, Internal jugular vein, Parotid gland, Carotid artery, Anatomical variation

## Abstract

**Purpose:**

The occipital artery (OA), typically arising from the external carotid artery (ECA), usually courses deep to the posterior belly of the digastric muscle as it ascends toward the skull base. While variations in the height of origin from the ECA are known, aberrant courses of the OA have not been previously documented.

**Methods:**

This study presents a four-case series identified through retrospective analysis of archived CT angiograms involving three female and one male patient, aged between 57 and 66.

**Results:**

In two cases, the OA was observed to pass deep to the internal jugular vein instead of its usual superficial trajectory; these variants were located on the left side. In the remaining two cases, the OA demonstrated aberrant retromandibular courses deep within the parotid glands. One of these cases revealed bilateral retromandibular segments of the OA, while the other exhibited a common occipitoauricular trunk originating from the right superficial temporal artery within the parotid space, which subsequently divided into the posterior auricular artery and the OA.

**Conclusion:**

These previously unreported anatomical variations are clinically significant and should be considered during surgical or interventional procedures involving the parotid region.

## Introduction

The external (ECA) and internal (ICA) carotid arteries typically arise from the carotid bifurcation within the carotid triangle. The ECA further supplies the superior thyroid (STA), lingual (LA), facial (FA), occipital (OA), ascending pharyngeal (APA) and posterior auricular (PAA) arteries and terminates with the maxillary and superficial temporal arteries.

The OA arises in the neck from the ECA and runs towards a groove medial to the mastoid process [13]. It may leave the ECA forming a common occipitoauricular trunk (OAT) with the PAA [9]. The OA can also arise from the APA, stylomastoid artery, or posterior meningeal branches.

The OA typically leaves the ECA at the level of the posterior belly of the digastric muscle (PBD) and courses deep to it towards the mastoid. In this course, it crosses over the internal jugular vein (IJV) [12]. However, the vertical level of the origin of the OA is variable, and in 23.33% of cases, it is above the gonial angle [12]. Acar et al. (2013) observed that the OA may originate from almost every level of the ECA [1].

The course of the OA deep to the IJV and its origin in the parotid space are aberrant topographic variants of the OA that we present here.

## Material and method

Four different cases were selected from a batch of 150 archived computed tomography angiograms. The first case was a 66-year-old male. The other three cases were females aged 57, 57, and 61. The research adhered to the principles of the World Medical Association’s Code of Ethics (Declaration of Helsinki). The responsible authorities (affiliation 2) approved the study (approval no.737/01 November 2024). The CT scans were performed using a 32-slice scanner (Siemens Multislice Perspective Scanner, Forcheim, Germany) with a 0.6 mm collimation and a reconstruction of 0.75 mm thickness, with 50% overlap, for a multiplanar maximum intensity projection. We used the Horos software for macOS (Horos Project), as in previous studies [4]. The findings were verified through two-dimensional reconstructions and three-dimensional volume renderings.

The angio-CT scans were initially acquired for clinical indications unrelated to thyroid, neck, or cervical vascular pathology, most commonly for the evaluation or follow-up of cerebral vascular conditions. High-quality angio-CT scans provided clear visualisation of anatomical structures. The carotid arteries and their branches were completely opacified, and the vertical course of the scans was complete for the head and neck. There were no pathological conditions that could distort the carotid anatomy.

All authors independently assessed the angio-CT scans. In cases where discrepancies arose between the assessments, the evaluation provided by the more experienced reviewer (M.C.R.) was considered definitive. Both reviewers were blinded to patient data and assessed the scans using the same standardised alignment to minimize bias.

## Results

In two female cases, aged 57 and 61, the OA was found coursing medially to the IJV. In the male case and another 57-year-old female, the OA coursed deep to the parotid gland (retromandibular course of the OA).

### The course of the OA deep to the IJV

**Fig. 1:**
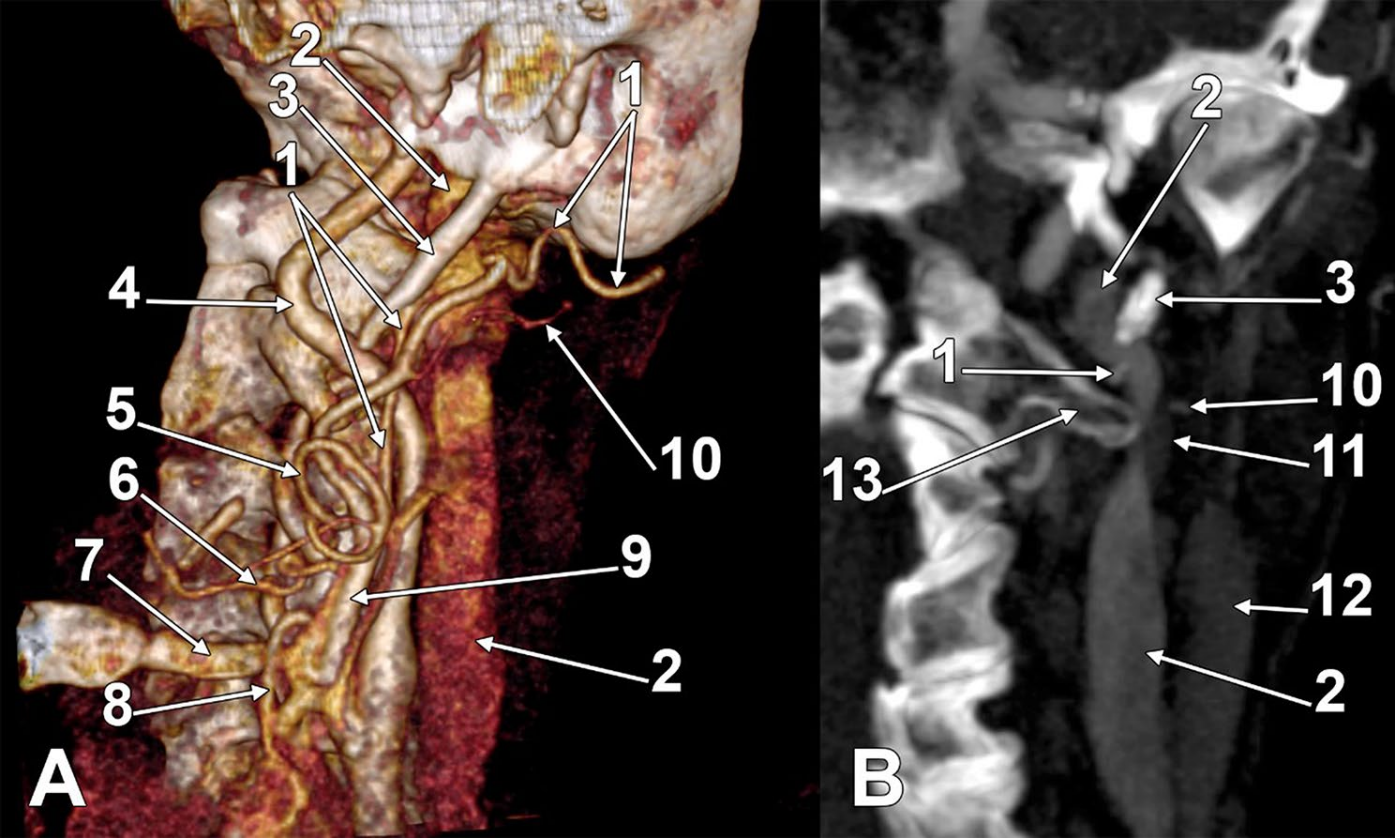
The left occipital artery’s course deep to the internal jugular vein. (**A**) Three-dimensional volume rendering, left lateral view. (**B**) Coronal slice through the left internal jugular vein, anterior view. (1) occipital artery; (2) internal jugular vein; (3) styloid process; (4) internal carotid artery; (5) facial artery; (6) lingual artery; (7) greater hyoid horn; (8) superior thyroid artery; (9) external carotid artery; (10) posterior auricular artery; 11. posterior belly of the digastric muscle; 12. sternocleidomastoid muscle; 13. transverse process of the atlas

In the first case (57-year-old female) (Fig. 1), the left internal jugular vein (IJV) appeared compressed against the transverse process of the atlas and, above it, by the styloid process. The left carotid bifurcation was 0.76 cm below the left greater hyoid horn. At the level of the greater hyoid horn, the STA left from the ECA and looped superiorly at 0.37 cm above the greater horn before descending to the thyroid lobe. The LA and FA left independently from the ECA. The OA originated from the posterior aspect of the ECA at 1.75 cm superior to the greater hyoid horn. It ascended 3.23 cm, crossing the ICA laterally and reaching the styloid process. Then, it turned posteriorly and crossed medially the IJV. At that level, the IJV was crossed laterally by the PBD and the PAA. Therefore, immediately above the transverse process of the atlas, the IJV had the OA on its medial side and the PAA on its lateral side.

**Fig. 2:**
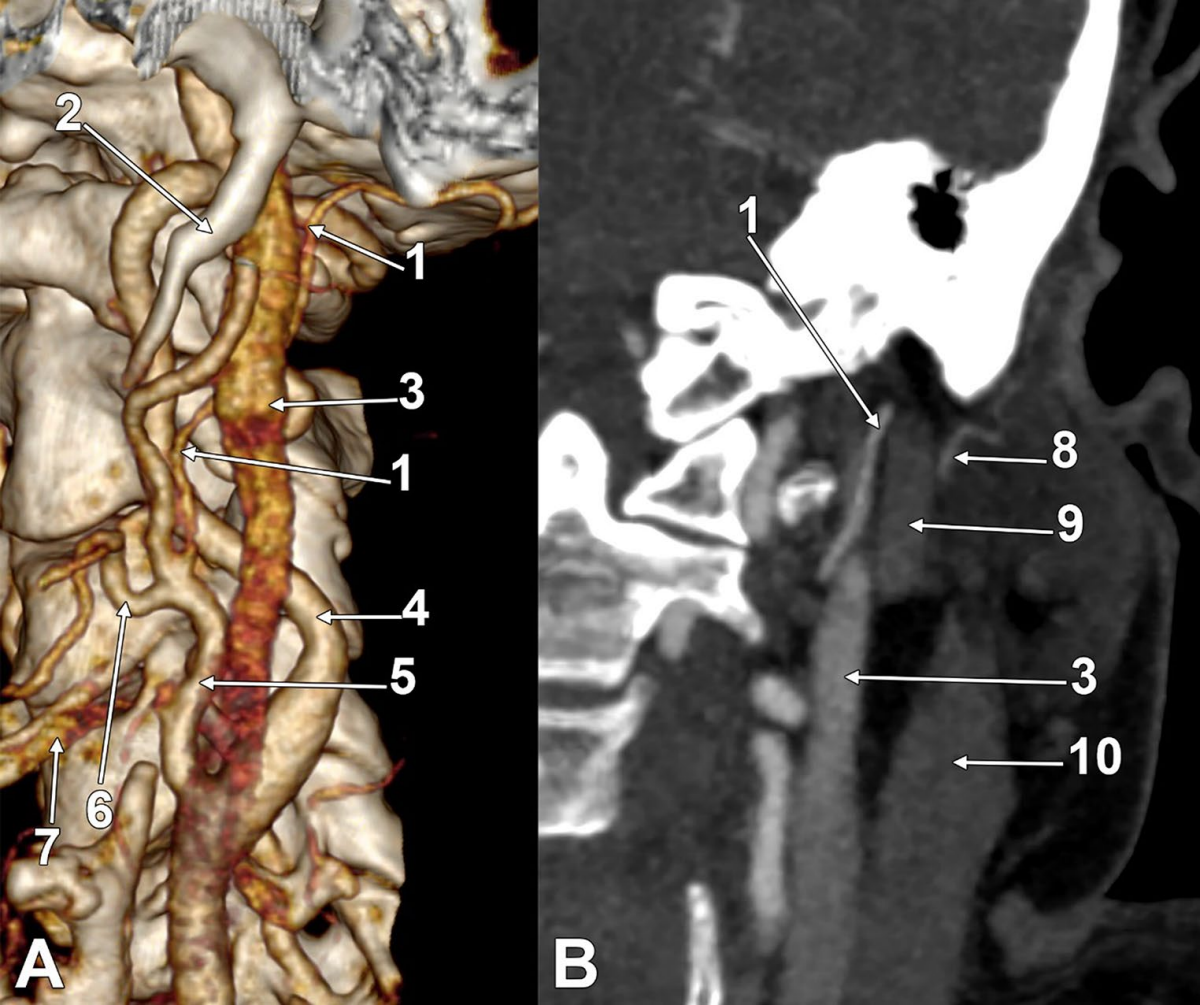
The left occipital artery’s course deep to the internal jugular vein. (**A**) Three-dimensional volume rendering, left antero-lateral view. (**B**) Coronal slice through the left internal jugular vein, anterior view. (1) occipital artery; (2) styloid process; (3) internal jugular vein; (4) internal carotid artery; (5) external jugular vein; (6) linguofacial trunk; (7) greater hyoid horn; (8) posterior auricular artery; (9) posterior belly of the digastric muscle; (10) sternocleidomastoid muscle

In the second case (61-year-old female) (Fig. 2), there was no compression of the left IJV against the transverse process of the atlas. The left carotid bifurcation was at the level of the upper border of the thyroid cartilage. A linguofacial trunk originated from the anterior aspect of the ECA at 0.57 cm above the greater hyoid horn. At the same level of the ECA, the OA left its posterior aspect. It ascended 1.36 cm and crossed over the ICA. Then, it continued postero-superiorly on the medial side of the IJV. After that „X” crossing, the OA continued on the posterior side of the IJV for 1.23 cm to reach the deep side of the PBD below the skull base. The PAA crossed the outer side of the PBD.

### The retromandibular segment of the OA

**Fig. 3:**
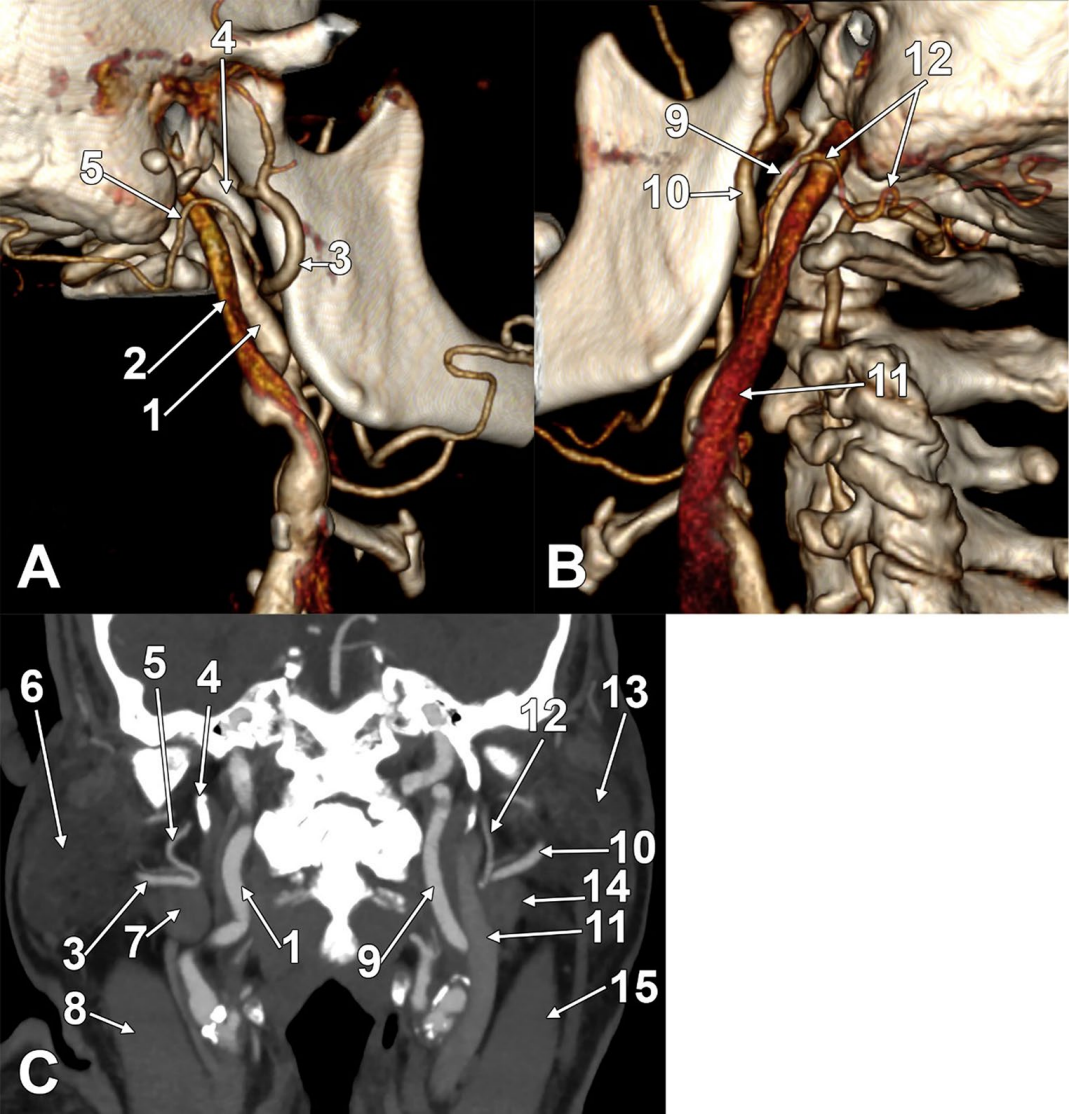
Bilateral retromandibular segments of the occipital arteries. **A**. Three-dimensional volume rendering, right side, lateral view. **B**. Three-dimensional volume rendering, left side, postero-lateral view. **C**. Coronal slice through the parotid glands, anterior view. (1) right internal carotid artery; (2) right internal jugular vein; (3) right external carotid artery; (4) right styloid process; (5) right occipital artery; (6) right parotid gland; (7) posterior belly of the right digastric muscle; (8) right sternocleidomastoid muscle; (9) left internal carotid artery; (10) left external carotid artery; 11. left internal jugular vein; 12. left occipital artery; 13. left parotid gland; 14. posterior belly of the left digastric muscle; 15. sternocleidomastoid muscle

In a 66-year-old male, bilateral aberrant retromandibular courses of the OAs were found (Fig. 3). Both carotid bifurcations were at the level of the hyoid bone. The branches of the ECAs had independent origins. On both sides, the ECAs had lateral retromandibular loops. On both sides, the OAs left the ECAs at the midheight of the mandibular ramus and had retromandibular superior loops deep to the parotid glands and lateral to the IJVs, thus reaching the PBD on that side.

**Fig. 4:**
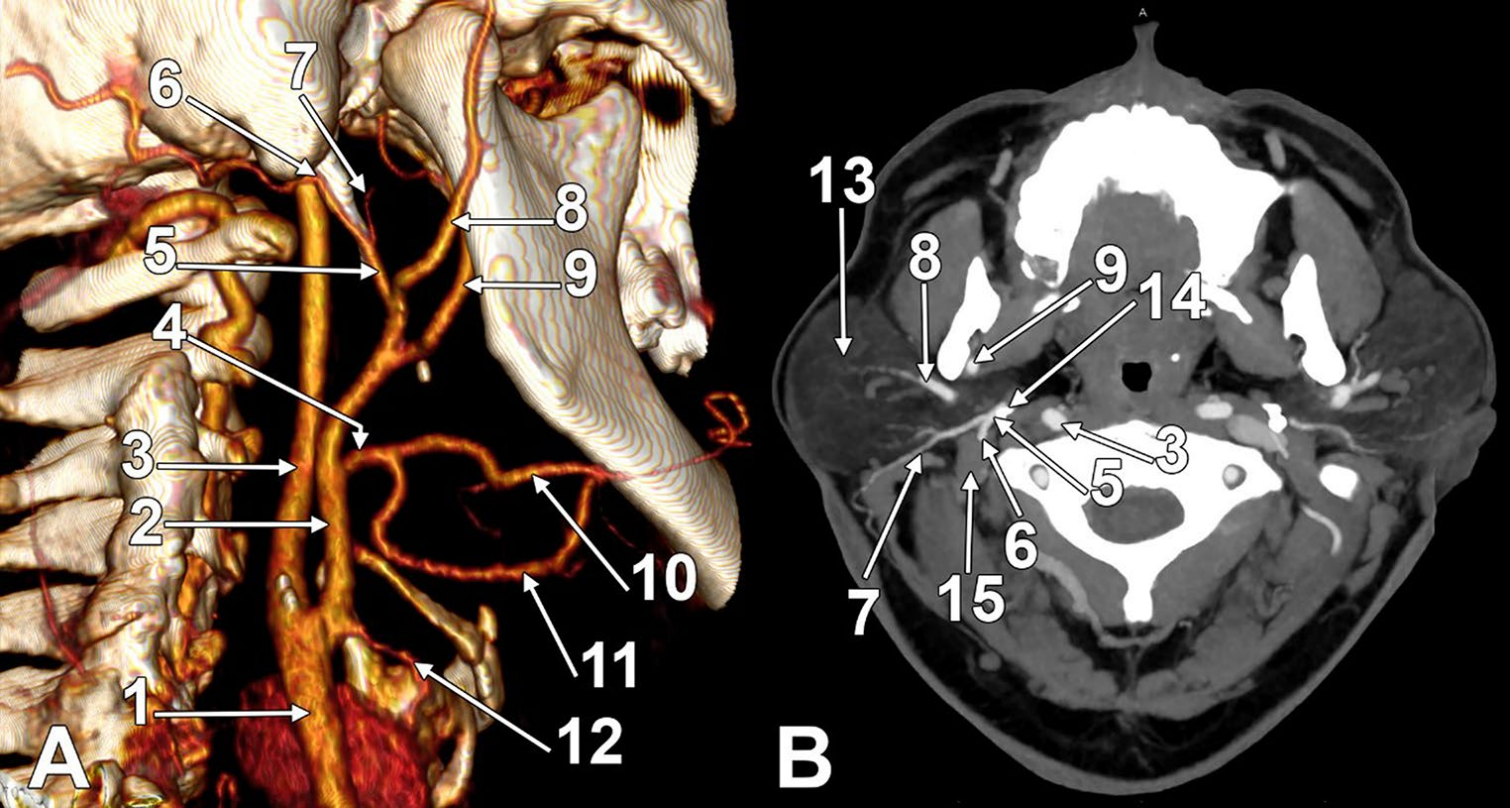
Left occipitoauricular trunk with retromandibular origin from the superficial temporal artery. **A**. Three-dimensional volume rendering, left lateral view. **B**. Axial slice through the parotid glands, inferior view. (1) common carotid artery; (2) external carotid artery; (3) internal carotid artery; (4) linguofacial trunk; (5) occipitoauricular trunk; (6) occipital artery; (7) posterior auricular artery; (8) superficial temporal artery; (9) maxillary artery; (10) facial artery; 11. lingual artery; 12. superior thyroid artery; 13. parotid gland; 14. styloid process; 15. posterior belly of the digastric muscle

In a 57-year-old female, only the right OA had an aberrant retromandibular origin and initial course (Fig. 4). The carotid bifurcation on that side was at the level of the upper border of the thyroid cartilage. A linguofacial trunk originated at 0.83 cm above the greater hyoid horn from the right ECA. The ECA entered the parotid space, deep to the parotid gland, and at 0.58 cm above the gonial angle, it divided into the maxillary and superficial temporal arteries. At 0.46 cm distally to its origin, the superficial temporal artery gave off a common occipitoauricular trunk that was 0.86 cm long. That trunk reached the lateral side of the styloid process’ tip and divided into the OA and PAA. The OA continued deep to the PBD.

## Discussion

Detailed knowledge of the anatomy of the OA is of utmost importance to avoid potential disastrous complications during therapeutic embolisation of neoplastic or vascular processes fed by the OA [2]. Knowledge of the course and diameter of the OA is also important because the OA is used as a donor vessel in extra-to-intracranial bypasses to the cerebral circulation [2]. As a companion vessel of the PBD, the OA crosses usually over the IJV. The OA does not have a constant vertical level of origin from the ECA, nor a single landmark for a safe identification [3]. To our knowledge, the course of the OA deep to the IJV was not reported previously. However, when the suboccipital segment of the OA is approached, such as in vertebral artery revascularization [7], this modified course may not be of great relevance. The OA can be involved in dural arteriovenous fistulas and is a feasible artery for the embolisation of these [6]. True aneurysms and pseudoaneurysms can develop in the OA and may be managed through either surgical resection or endovascular embolization [6]. Vascular lesions are a significant but often overlooked cause of parotid region masses [10]. An intraparotid OA represents a novel, yet plausible site for such vascular pathologies, and should be included in the differential diagnosis of parotid swellings.

OA pseudoaneurysm secondary to trauma during radical neck dissection is rare but potentially life-threatening, usually presenting as a progressively enlarging pulsatile mass [14]. When the OA originates within the parotid gland, as in our case, a pseudoaneurysm may also occur as a complication following parotid surgery. This anatomical variation increases the risk of iatrogenic vascular injury and may necessitate urgent endovascular embolization to prevent hemorrhagic or neurologic complications. Surgeons should be aware of this possibility, particularly in cases involving unexpected vascular structures within the parotid space.

The OA can also be used for intraoperative angiography [6]. On the other hand, when the carotid triangle is surgically dissected, an OA running beneath the IJV may appear as a falsely absent OA. The distinction between true and false OAs may be determined through imaging examinations. It has also been reported that the sternomastoid branch of the occipital artery (SBOA) demonstrates a consistent size and location within the reflected fascia of the sternomastoid muscle [11]. Notably, the spinal accessory nerve was within 11 mm of the SBOA in 100% of cases [11]. However, in cases where the OA has intraparotid origin, this anatomical variation may compromise the reliability of the SBOA as a visual landmark for locating the spinal accessory nerve, thereby increasing the risk of nerve injury during neck dissection. Surgeons may misidentify small muscular branches or accessory arteries as the SBOA, potentially leading to disorientation and heightened risk during surgical navigation.

Successive branches of the ECA may form various common trunks of origin, such as the thyrolingual, thyrolinguofacial, linguofacial, and occipitoauricular trunks [8, 15]. They must be carefully distinguished when approaching the carotid axis.

Although the LFT was reported consistently, a recent study demonstrated that its morphology and topography are individually variable and should be evaluated on a case-by-case basis [5]. The unilateral linguofacial trunk was found to have a prevalence of 15.38% [15]. This variant was identified in 2 out of 4 cases reported here.

The occipitoauricular trunk occurs in 14% of cases [8]. This report describes a variant that differs from previously documented cases. Specifically, it originates above the PBD, deep to the parotid gland, and arises from the superficial temporal artery rather than the ECA, representing a novel anatomical variation. Consequently, during dissection of the intraparotid portion of the superficial temporal artery, caution should be exercised to identify a possible aberrant origin of an occipitoauricular trunk.

## Data Availability

No datasets were generated or analysed during the current study.
